# Open Label Trial of the Tolerability and Efficacy of Zonisamide in the Treatment of Alcohol Dependence

**DOI:** 10.3109/00952991003674812

**Published:** 2010-03-25

**Authors:** Clifford M. Knapp, Ofra Sarid-Segal, Mark A. Richardson, Laurie Sickles Colaneri, Maryam Afshar, Eric Devine, Chris C. Streeter, Joanne Piechniczek-Buczek, Domenic A. Ciraulo

**Affiliations:** ^a^ *Division of Psychiatry, Boston University School of Medicine, Boston, Massachusetts, USA*

**Keywords:** Alcoholism, anticonvulsant, clinical trial, neuropsychological tests

## Abstract

***Objectives*: The objectives of this study are to assess the tolerability and efficacy of the anticonvulsant zonisamide in an open label trial of the treatment of alcohol dependence. *Methods:* In this trial, zonisamide (400-mg daily) was administered to alcohol-dependent subjects (ADS) (*n* = 16) over 13 weeks. The mean daily consumption of standard alcoholic drinks and performance on a verbal fluency task, the COWAT, and on a measure of attention and visuomotor speed, the DSMT were assessed, and the occurrence of adverse events was monitored weekly. *Results:* The mean number of drinks consumed daily was significantly reduced from baseline levels during the treatment period. Performances on the COWAT and on the DSMT were not significantly reduced by zonisamide treatment. Overall, zonisamide was well tolerated by the study subjects. *Conclusion*: These results indicate that zonisamide administration may not impair verbal fluency in ADS, and are consistent with other studies that found zonisamide administration may reduce alcohol intake.**

## INTRODUCTION

The anticonvulsant agent topiramate has been shown in placebo-controlled clinical trials to reduce heavy drinking in alcohol dependent individuals (1, 2). Zonisamide, which has some structural similarity to topiramate (3), may have similar efficacy in the treatment of alcoholism. This drug has been shown to reduce alcohol consumption in mice and rats (4). Individuals with a history of high risk drinking decreased the amount of ethanol self-administered when treated with a single dose of zonisamide from levels seen with placebo administration (5).

Following one year of zonisamide treatment, deficits in word recall and verbal fluency have been observed in seizure patients (6). Verbal fluency has been found to be impaired by other anticonvulsant agents, including topiramate, in healthy subjects and seizure disorder patients (7, 8). Thus, there is reason for concern as to how this drug might alter verbal fluency in alcoholic patient populations.

In the present open label trial zonisamide was administered to alcohol dependent subjects to determine its tolerability and its efficacy in reducing ethanol use as a prelude to its use in a large clinical trial. Two neuropsychological tests were administered to subjects in this trial that had revealed topiramate-induced deficits in cognitive functioning in a prior clinical trial in alcohol dependent subjects (9). These tests were the Controlled Word Association Test (COWAT), which assesses verbal fluency (10) and the other, the Digit Symbol Modalities Test (DSMT), which measures visuomotor speed and sustained attention (11).

## METHODS

The study described here was conducted with the approval of the Boston University Medical Center's Institutional Review Board. Study candidates were required to provide informed written consent prior to being allowed to participate in this study.

This was an open label clinical trial in which the zonisamide was administered over a period of 13 weeks. Treatment-seeking men and women had to be between 21 to 64 years old to enter this study and had to meet criteria for a DSM-IV-TR diagnosis of alcohol dependence (12). The number of standard alcoholic drinks consumed per day by subjects was assessed using the Time-Line Follow Back (TLFB) method (13). Minimal requirements for alcohol consumption were 14 drinks per week for women and 21 drinks per week for men, over a 28 consecutive day period during the 90 days preceding screening, and Alcohol Use Disorders Identification Test (AUDIT) scores needed to be greater than 8 (14). Dependence on substances other than alcohol, caffeine, and nicotine, as determined using the Structured Clinical Interview for DSM-IV-TR, and confirmed by urine toxicological screens, was one of the exclusion criteria for this trial.

The dose of zonisamide was gradually increased to a maximal target dose of 400-mg per day by Week 7. This dose is in the range of the higher doses of zonisamide used to treat seizure disorders (15).

Subjects were maintained on their maximal dose for five weeks after which the dose was reduced over the last two treatment weeks. The target dose and the rate of dosage increase could be reduced by the study physician if needed to a level tolerated by the subject. As an adjunct to medication treatment, subjects attended weekly 20 to 30 minute therapy sessions in which BRENDA guidelines were followed (16).

Subjects were evaluated with the following assessment tools at baseline: Clinical Institute Withdrawal Assessment of Alcohol Scale, Revised (CIWA-Ar) (17), HAM-D-17 (18), HAM-A (19), Obsessive Compulsive Drinking Scale (OCDS) (20), Michigan Alcohol Screening Test (MAST) (21), Alcohol Dependence Scale (ADS) (22), Medical Outcomes Sleep Scale (MOS) (22), COWAT, and DSMT. The CIWA-AR was then administered on a weekly basis. Queries concerning self-reports of adverse events were made on a weekly basis. The COWAT, DSMT, Clinical Global Impression (CGI)-observer/self report scales, HAM-A, HAM-D, and the OCDS also were to be obtained on Treatment Weeks 4, 8, and 12. In some cases these measures were collected on the next week if a subject failed to attend a scheduled visit.

Data obtained in this study were analyzed using an intent-to-treat approach. Data were evaluated using a repeated measures mixed models analysis with treatment time as a within subject factor (23).

## RESULTS

Demographic information, prescreening alcohol consumption levels, and mean ADS, AUDIT, and MAST scores for subjects are shown in Table 1. Sixteen subjects were entered into this trial. Nine subjects completed all 13 weeks of treatment. Four subjects left the study after completing either only one or two weeks of treatment. Ten subjects entered the maintenance phase of this study. The mean daily maintenance dose was 390 (SE ± 10) mg for these subjects, with only one subject not being able to tolerate the 400-mg daily dose.

**TABLE 1.** Demographic and clinical characteristics of alcohol dependent subjects admitted into the zonisamide trial. Mean (± SE) values are presented where appropriate.

	Zonisamide (*n* = 16)
Age	50.7 ± 1.6
Sex	
Male	13
Female	3
Percent Caucasian	100
Education (yrs)	16.0 ± 1.1
Pre-screening drinks/day	10.4 ± 1.5
ADS	14.1 ± 1.5
AUDIT	23.6 ± 1.2
MAST	7.7 ±.9

On the study entrance day, i.e., the baseline visit, a mean CIWA-AR score of 4.1 (±.7) was obtained for subjects. There was a significant decrease [F(13,115) = 2.5; *p* = .005] in CIWA-AR scores over the course of the treatment period with the lowest mean score of 1.1 (±.3) being reached on treatment Week 10. These CIWA-AR scores are not indicative of clinically significant withdrawal. Mean HAM-D scores declined from a baseline value of 6.9 (± 1.0) to 4.6 (± 1.8) by Week 12. The values are below a score of 10, a score, which can be considered to be indicative of mild depression. One subject, however, received a score of 18 during Week 5, which may reflect moderate to severe depression. HAM-A scores declined from a mean baseline value of 6.1 (± 1.0) to a value of 3.2 (± 1.0) for Week 12, with a score of 18 and above on the Ham-A being consistent with the presence of severe to moderate anxiety.

The mean number of standard drinks consumed daily in the baseline interval (i.e., the interval between screening and the beginning of treatment) was 8.8 (± 1.6). This value declined to 2.3 (±.8) and 2.1 (± 1.4) for treatment Weeks 8 and 12, respectively. The time effect was significant [F(13, 110) = 3.3; *p* = .0003] for the mean daily number of alcoholic drinks consumed for each week. The time effect in subjects’ OCDS scores was found to be significant [F(3,28.8) = 4.9; *p* = .007] with mean scores declining from 25.3 (± 1.2) for the baseline visit to 19.3 (± 3.1) for Week 12. CGI clinician ratings of “severity of illness” went from 4.1 (±.2) for the baseline visit to 2.4 (±.5) for Week 12. The time effect for this measure was significant [F(3,31.9) = 9.56; *p* = .0001]. The time effect was also significant [F(3, 30.4) = 8.0; *p* = .0004] for the self-rated severity on the CGI.

Mean scores for performance on the COWAT and DSMT are presented in Table 2. Within subject comparisons failed to show significant changes in these values over the treatment period. The Mean MOS scores for Sleep Disturbance at the baseline visit was 36.3 (± 5.0). This was somewhat higher than the mean value of 24.5 reported for the general population (22). The Sleep Disturbance score was decreased to a mean of 27.0 (± 6.7) by Week 12, but the difference between this value and the baseline value was not significant.

**TABLE 2.** Mean (± SE) and (range) of values obtained for the effects of zonisamide on the phonetic and semantic portions of the Controlled Word Association Test (COWAT) and on the Symbol Digit Modalities Test (DSMT).

Treatment Week	Week 0	Week 4	Week 12
COWAT semantic	21.1 (± 1.4) (11–32)	21.5 (± 1.4) (14–32)	21.9 (± 2.0) (16–29)
COWAT phonetic	45. 3 (± 3.9) (21–65)	37.0 (± 3.9) (24–67)	38.9 (± 3.9) (22–58)
DMST	53.3 (± 3.0) (38–65)	48.9 (± 3.0) (33–64)	51.8 (± 4.6) (44–79)

One subject experienced severe adverse events that were probably related to zonisamide administration. These consisted of sadness and tearfulness. This subject also experienced moderate adverse events that consisted of somnolence and disinhibition. Two other subjects experienced sedation that was rated as a moderate adverse event that was probably related to treatment with zonisamide.

## DISCUSSION

The significant reduction in alcohol consumption observed during the treatment period in this study is consistent with the notion that zonisamide may have efficacy as a medication for the treatment of alcohol dependence. Other evidence of the efficacy of zonisamide included significant reductions in both self- and clinician ratings of “severity of illness” on the CGI scales. In addition, alcohol craving as assessed by the OCDS was significantly reduced from baseline levels during treatment with zonisamide. These findings are in accord with preliminary results from a recent clinical trial in which zonisamide administration reduced ethanol consumption to a greater degree than did placebo in dependent subjects (24). These results were for a small number of subjects with nine subjects receiving zonisamide and nine placebo with the difference between groups in changes in alcohol consumption approaching significance. Another difference between the current study and the study of Arias et al. is that it does not appear that Arias et al. used formal neuropsychological tests.

Subjects treated with zonisamide in the present study did not exhibit significant changes in performance on a test of attention, visual scanning, and psychomotor speed, or on a test that measures verbal fluency. Although not significant, there was a 14 to18% decrease in mean performance levels on the phonetic portion of the COWAT during the treatment period. This can be compared to 30 and 39% lowering of performance produced by topiramate treatment in this measure seen in alcoholic (9) and healthy (8) subjects, respectively.

Overall, zonisamide was well tolerated by subjects in the present study with only one subject in the maintenance phase not able to receive the 400-mg daily dose. Zonisamide administration was not associated with marked elevations in the mean values obtained for measures of anxiety, mood, or sleep disturbance during a period in which subjects’ alcohol use was decreased. One subject, however, did experience severe symptoms of sadness while receiving zonisamide.

Limits of this study include lack of inclusion of a placebo control and the use of only one target dose. The findings presented here suggest that in alcohol dependent individuals, zonisamide, at a dose used for maintenance therapy in seizure disorders, does not produce significant impairment of verbal fluency or visuomotor speed and sustained attention as assessed by the DSMT. This investigation, however, has only a limited power to detect such impairments because of the small number of subjects included in the study. Overall, the findings of this investigation are consistent with the idea that zonisamide may have efficacy in the treatment of alcohol dependence.

## Declaration of Interest

Drs. Domenic A. Ciraulo, Maryam Afshar, and Ofra Sarid-Segal have received research support from Alkermes, Bristol Myers Squibb, Catalyst Pharmaceutical Partners, Drug Abuse Sciences, Janssen, Lipha, Orhto McNeil, and UCB Pharma. Dr. Domenic A. Ciraulo has received consulting fees from Alkermes, Bristol Myers Squibb, Janssen, Ortho McNeil, Organon, and Trancept. Dr. Joanne Piechniczek-Buczek has received research support from Bristol Myers Squibb, Catalyst Pharmaceutical Partners, Ortho McNeil, and UCB Pharma.
